# Translational Application of a Neuro-Scientific Multi-Modal Approach Into Forensic Psychiatric Evaluation: Why and How?

**DOI:** 10.3389/fpsyt.2021.597918

**Published:** 2021-02-05

**Authors:** Cristina Scarpazza, Alessio Miolla, Ilaria Zampieri, Giulia Melis, Giuseppe Sartori, Stefano Ferracuti, Pietro Pietrini

**Affiliations:** ^1^Department of General Psychology, University of Padova, Padova, Italy; ^2^Department of Psychosis Studies, Institute of Psychiatry, Psychology and Neuroscience, King's College London, London, United Kingdom; ^3^Molecular Mind Laboratory, IMT School for Advanced Studies Lucca, Lucca, Italy; ^4^Department of Human Neurosciences, “Sapienza” University of Rome, Rome, Italy

**Keywords:** insanity evaluation, cognitive bias, neurocriminology, forensic psychiatry, neuroethics

## Abstract

A prominent body of literature indicates that insanity evaluations, which are intended to provide influential expert reports for judges to reach a decision “*beyond any reasonable doubt*,” suffer from a low inter-rater reliability. This paper reviews the limitations of the classical approach to insanity evaluation and the criticisms to the introduction of neuro-scientific approach in court. Here, we explain why in our opinion these criticisms, that seriously hamper the translational implementation of neuroscience into the forensic setting, do not survive scientific scrutiny. Moreover, we discuss how the neuro-scientific multimodal approach may improve the inter-rater reliability in insanity evaluation. Critically, neuroscience does not aim to introduce a brain-based concept of insanity. Indeed, criteria for responsibility and insanity are and should remain clinical. Rather, following the falsificationist approach and the convergence of evidence principle, the neuro-scientific multimodal approach is being proposed as a way to improve reliability of insanity evaluation and to mitigate the influence of cognitive biases on the formulation of insanity opinions, with the final aim to reduce errors and controversies.

## Introduction

Recent findings from neuroscience research challenge traditional approaches to different aspects of forensic evaluations. The use of neuro-scientific evidence in criminal proceedings has increased tremendously in the United States over the last two decades (1). The most critical topic of discussion regards the relevance of neuroscience in insanity evaluations. Most legal systems consider mental insanity as reason to mitigate or exclude responsibility in the case of individuals affected by a mental illness that greatly reduced or abolished their capability for self-determination at the time of the crime (2, 3).

The typical approach to mental insanity evaluation is based mostly only on unstructured psychiatric interviews with the defendant. Though semi-structured interviews may also be utilized in a mental insanity assessment, in some countries, including Italy, their use is often regarded as superfluous by experienced clinicians. A limitation of both unstructured and structured interviews is that they rely to a considerable extent on the defendant self-reports (3).

A growing body of data suggests that criminal behavior has a multifaceted neurobiological component, including genetic and epigenetic factors as well as structural and functional brain correlates (4–12). These novel pieces of knowledge have intensified judicial interest in the potential application of neuroscience to criminal law (13). The so-called “neuro-scientific” approach aims to complement the classical approach to mental insanity evaluation by providing objective indices of possible brain function alterations. To this aim, the neuro-scientific approach requires that multiple pieces of evidence from different disciplines coherently converge to support an hypothesis (14). Neuropsychological assessments and reaction time based behavioral tasks are two examples of methods that are considered to be neuro-scientific, as they provide an objective evidence of brain functioning and go beyond the patient's self report. For this reason, the neuro-scientific approach is considered to be trans-disciplinary and multimodal. Furthermore, according with the neuro-scientific approach, insanity evaluations in specific cases may be integrated by instrumental analyses, including magnetic resonance imaging (MRI), positron emission tomography (PET) and other methods (15).

On one hand, neuroscientists believe that insanity assessments should be enriched by adopting a multimodal trans-disciplinary approach that provides an objective indirect evidence of how the brain functions. Neuro-scientific techniques are thought to increase the reliability of the evaluation (4, 16, 17) as they are less prone to any (un)conscious exaggeration of symptoms for defensive purposes as well as to human cognitive biases. On the other hand, this approach has been heavily criticized by other authors (18–21), who consider psychiatric interviews sufficient to evaluate the defendant mental state. Neuroscientists who support the use of a multimodal approach in criminal trials do not deny that the legal criteria for insanity are, and should remain, based on the evaluation of the individual behavior (15, 22). However, they also sustain that the clinical evaluation, based on unstructured interviews only, often leads to subjective and inconclusive opinions on insanity. Furthermore, though the results of insanity evaluations are intrinsically probabilistic inferences, this covert diagnostic uncertainty is not translated into a probabilistic decision (“The defendant is insane with 60% of probability”), but rather in a categorical one (sane, partially insane, totally insane). These categories may vary across different countries (23), further complicating the situation. In criminal trials, judges and jurors should reach their decisions in accordance with the “*beyond any reasonable doubt*” (BARD) principle. Indeed, this principle was meant to prevent the outcome of a trial from being influenced by subjectivity and arbitrariness. Judge and juror decisions should be rational, clearly justified and based on reliable scientific evidence. However, their decisions are often influenced by the results of the insanity evaluations which, in turn, are themselves disputable (24).

The aim of the neuro-scientific approach is not to change the way insanity is defined, but rather to improve the reliability of insanity assessments, mitigate the potential influence of cognitive biases and reduce controversies in criminal cases. Thus, in cases in which, using the typical approach to insanity, different experts reach different conclusions, a multimodal trans-disciplinary approach can provide objective data crucial to disentangle whether or not objective measures of psychopathology are present and clinically relevant. Furthermore, if different independent pieces of evidence converge toward an opinion on insanity, it would become very difficult to support the opposite conclusion without falling into logical biases. In the following sections, we will discuss the reasons that make the classical approach to insanity defense biased by low inter-rater reliability. We also will explain why we believe that criticisms to the adoption of multimodal neuro-scientific methods to complement insanity evaluation are not scientifically supported. Finally, we will discuss how a multimodal trans-disciplinary approach can improve insanity evaluation procedures.

## The Intrinsic Low Reliability of Clinical Assessment of Mental Disorders and Insanity

### Psychiatric Evaluations Using Unstructured Interviews Only Are Characterized by a Low Inter-rater Reliability

Insanity evaluation requires the assessment of psychiatric conditions that can greatly diminish or completely abolish the ability to understand and/or to freely will and to establish a causal link between the abnormal mental condition and the criminal act (23). The first part of this process is characterized by a low inter-rater reliability even in the clinical setting. Reliability of the diagnostic process in psychiatry is greatly challenged by the absence of biomarkers (25, 26), that is, features that can be objectively measured and are indicative of normal or pathological processes (27). Second, the underlying brain pathology is not evident at visual inspection of brain scans, but may only be identified through complex analyses of brain imaging data (28). Third, many psychopathological symptoms are common to different disorders (29). For these reasons, the diagnostic criteria are created on consensus and are therefore questionable. For instance, one of the criteria for schizophrenia requires that “*For a significant portion of time since the onset of the disturbance, level of functioning in one or more major areas (...) is markedly below the level achieved prior to the onset*”. It is not clear what “*significant portion of time*” means. In the forensic setting, this leaves the criterium open to different interpretations and controversies. Thus, psychiatric assessments based on unstructured interviews only suffer from low inter-rater reliability (30–32), even when experienced psychiatrists are involved [for instance, in (31, 33) raters have more than 20 years of clinical experience]. In other words, different clinicians who examine the same patient are likely to reach different diagnostic conclusions. That is what can be observed on a daily basis not only in the clinical field, but even more so in the forensic context. Inter-rater reliability is measured by an index (kappa) that ranges from 0 to 1, where 0 refers to absence of agreement and 1 to complete agreement among different raters. For instance, DSM-5 fields trial research reported that the inter-rater agreement for schizophrenia was 0.46, while the inter-rater agreement for major depression was only 0.28 (32). Importantly, the same problem is also present in forensic setting (34).

### Insanity Evaluation Is Characterized by a Low Inter-rater Reliability

Insanity evaluation requires to determine (1) whether the defendant is affected by a psychiatric disorder; (2) whether and to what extent that disorder also affects the defendant's ability to understand and/or to freely will; and (3) whether there is a causal link between the mental condition and the criminal act. A recent systematic review and meta-analysis of previous studies that investigated inter-rater reliability of sanity opinions revealed agreement rates ranging from 57 to 100% and kappas ranging from 0.28 to 1.0 (24). The meta-analysis returned estimates of 0.41 (95% CI: 0.29–0.53) for sanity opinions. While this would be considered relatively strong from a mere statistical point of view, such a concordance rate is actually quite unsatisfactory when applied to the legal context, in which judges are required to reach decisions in compliance with the BARD principle.

The required evaluation of the existence of a causal link between the diagnosed mental condition and the committed criminal act may also be a factor of further discrepancy among experts. Specifically, causal link means that the crime must be an evident consequence of the underlying mental disorder: for instance, a causal link may be proven if an individual with a persecutory delusion hits a neighbor who the subject was convinced was spying on him, but not if the same individual steals a jacket from a store. The identification of this causal link is subjected to arbitrariness as, in the disregard of respect to accepted guidelines, some experts may detect a causal link, whereas others may not. Recently, the European Psychiatric Association (EPA) found that the evidence base for forensic-psychiatric practice is weak and therefore has provided recommendations for best practice (35). Moreover, the American Academy of Psychiatry and Law (AAPL) has published guidelines based on a comprehensive review of legal and psychiatric factors to be taken into account to perform insanity defense evaluations (Journal of AAPL, 2014). Finally, a recent work proposed an insanity assessment support scale based on clinical criteria (36). This instrument, however, still has to be validated. Overall, the adherence to standardized guidelines to fulfill shared and validated criteria to assess mental insanity remains to date largely unsatisfactory.

A recent retrospective study on insanity evaluations found that criminals judged as insane presented with more severe psychiatric symptoms and had a higher number of psychiatric treatments and involuntary hospitalizations as compared to those who ended up to be considered not mentally insane (37). In addition, time-bias (37) or crime-related (38) factors, more than strictly mental state related factors, appear to influence insanity opinions.

It should be emphasized that forensic psychiatric evaluation is a decision-making process in which a clinical, naturalistic element (the nosografic diagnosis) is unavoidably coupled with a juridical element (the expert's opinion on insanity). Insanity, indeed, is not a natural data *per se*; as a matter of fact, the criteria for insanity stated by the law, as well as other social factors, may lead to completely different conclusions in the same case, depending on the country where the evaluation is performed (39). This makes the comparison of procedures and results across different countries very difficult, further complicating the scientific evaluation of the field.

### Clinical Diagnosis Is Affected by Cognitive Fallacies/Biases

As any human being, also forensic experts may be vulnerable to cognitive fallacies. Cognitive fallacies are cognitive failures that subconsciously influence the perception and evaluation of evidence (40, 41). Cognitive fallacies are present event in disciplines in which mistakes are supposed to be essentially absent, including fingerprints and firearms analyses (41, 42) The fascinating research on cognitive biases in forensic sciences is rapidly expanding (40, 43–47), as well as their occurrence in real cases (14, 16). The first and most important result from these studies is the general lack of acceptance of the need for procedures to minimize cognitive biases and a failure to recognize susceptibility to biases (48), an effect that hereafter will be called “*denial cognitive bias*.” A similar bias is called “*blind spot*,” which refers to the tendency to recognize bias in others while remaining oblivious to one's own biases. Critically, the denial cognitive biases and the blind spot are the lesser acknowledged biases among forensic experts (49). Another predominant cognitive bias emerging in criminal trials, as revealed by a systematic review (43) and a survey (40), is the *confirmation bias*, consisting in the tendency to search for, agree with or interpret information in a way that confirms one's own presumptions (47). This cognitive bias is present in the great majority of criminal trials (16, 40, 42, 43, 50). In addition, forensic science is also affected by the *adversarial allegiance effect*, that is, the tendency to find pieces of evidence supporting the side that retains the expert (43, 51). Despite some legal systems, such as the Italian one, attempted to overcome this limitation by including a *super-partes* expert summoned by the judge (*Peritus*), this strategy alone is not sufficient to mitigate biases, as the *Peritus* also is subjected to subconscious biases, such as prejudice, denial bias, confirmatory bias and so on and so forth.

Intriguingly, cognitive biases can affect the observation of evidence, that is, the clinician's ability to detect psychiatric symptoms as well as the conclusions, defined as the cognitive interpretations of observations, that is, the psychiatric diagnosis (14, 44–46). As an example of bias at the level of the observation, Table 1 reports the symptoms observed by three different clinicians in a recent criminal case of a woman charged for supposedly killing her own husband and with previous documentation supporting the presence of chronic alcoholism (which, in Italy, is a relevant factor for insanity evaluation): three clinicians observed three different psychiatric symptom constellations Importantly, because he did not notice any signs or symptoms of chronic alcoholism, the *Peritus* denied additional investigations (neuropsychological test and an MRI) to verify the potential presence of neurological damages due to chronic alcoholism and eventually he concluded for the woman to be mentally sane.

**Table 1:** Observation of clinical symptoms by three different experts.

**Clinician n.1- Super partes expert – *Peritus***	**Clinician n.2 – Prosecutor consultant**	**Clinician n.3 –Defense consultant**
Depressed facies	Alcoholic facies	Paranoid ideas
Slovenly aspect	Absence of overt emotions	Bizarre delusions
	Absence of logical connections	Alcoholic facies
	Absence of self criticism	Slurred speech
	Memory deficit	Memory deficit

*This is a representative case of a woman prosecuted for killing her husband and evaluated by a court appointed expert (Peritus) and experts from the two adversarial sides*.

Furthermore, a representative example of cognitive bias in inter-rater reliability emerging at the level of the conclusions pertains to the famous Breivik case, in which different clinicians who observed the same symptoms reached very different diagnostic conclusions: specifically, narcissistic personality disorder combined with pseudologia fantastica as opposed to paranoid schizophrenia, obviously with major different implications for insanity (52). Another example of bias at the level of the conclusions was described in a recent paper, where self-reported information from the defendant were considered, respectively, either indicative of psychiatric symptoms or deprived of any significant meaning by different consultants (53).

### The Identification of Malingering Using Clinical Interview Only Is Very Challenging

An additional factor that makes forensic psychiatric evaluations extremely challenging is that defendants may intentionally malinger or exaggerate symptoms for defensive purposes (54). The malingering assessment is undoubtedly of primary importance in forensic settings. Nevertheless, some clinicians may perform this assessment inappropriately due to the so called Dunning-Kruger effect, a cognitive bias consisting in overestimating one's own competence in a specific topic (55). In contrast, scientific research indicates that experienced individuals, including judges, psychiatrists and forensic consultants, in detecting deception perform only slightly better than chance (56). In general, professional lie catchers exhibit accuracy rates in the range from 45 to 60%, with a mean of 54% (57, 58). In this regard, results from two well-known studies prompt important considerations. In a 1973 study (59), “pseudo patients” feigning hallucinations were all admitted to the psychiatric department of twelve different highly specialized hospitals: all but one (who was diagnosed with bipolar disorder) received a clinical diagnosis of schizophrenia. It is relevant to highlight that this study was performed in 1973, thus prior to the adoption of the more stringent diagnostic criteria included in the most recent versions of the DSM. While these results should be considered with caution, their broader meaning appears to be still valid. Indeed, in a more recent study (60), experienced psychiatrists distinguished actors from real depressed patients during a clinical interview with an accuracy close to the chance level. Furthermore, the clinicians rated their confidence in their diagnoses as 6.5 out of 10 in the case of patients and 7.1 in the case of actors, showing that they were equally certain in either case. Because clinical interviews cannot be used to reliably detect malingerers, researchers have developed tests that can support clinicians to detect malingering of cognitive (61, 62) and psychiatric disorders (63–65). These cognitive tests have a high accuracy rate. For instance, a mouse tracking and a reaction time cognitive test achieved an accuracy up to 96% in distinguishing liars (malingering psychiatric symptoms) from truly depressed patients and truth-tellers (65).

To conclude, the challenge psychiatry faces may turn into vulnerability in the court of law (3). Indeed, a prominent body of literature suggests that using the classical approach, the outcome of the insanity evaluation, which is taken into account by judges to reach a decision “beyond any reasonable doubt,” is characterized by a high level of uncertainty. Thus, any innovation that can improve its reliability, though imperfect, should be welcomed. Unfortunately, criticisms of the multi-disciplinary approach often neglect the intrinsic unreliability of this discipline and how the results of insanity evaluations can be affected by unconscious logical/cognitive fallacies. To deny these problems is a cognitive bias *per se* (40, 48).

## Criticisms to the Neuro-Scientific Approach: Are They Scientifically Grounded? If Not, Why?

In order to mitigate the effects of subjective variability in insanity assessment, some forensic experts welcomed the use of the neuro-scientific multimodal methodology in court. Indeed, this approach allows to support the preliminary clinical diagnosis made through unstructured interviews with the results from semi-structured interviews; to support the clinical opinions on self-determination ability by using standardized neuropsychological tests; to unveil the presence of malingering by using reaction-time based instruments; to corroborate the genesis and the dynamic of the criminal act with neuroimaging and neurobiological data. At the same time, the introduction of this approach in the forensic context has been highly criticized by other scholars. In the following paragraphs, we will take into consideration the main criticisms that have been raised against the neuro-scientific approach. We will explain also why we believe that these criticisms lack of a scientific basis. Indeed, in our opinion they lay on wrong assumptions or are not supported by the most recent and authoritative literature. The subheadings of the following paragraphs reflect the main criticisms.

### Neuroscientists Believe That Neuro-Scientific Methods (in Particular Neuroimaging) Alone Are Enough to Determine Insanity

“*The most pernicious error here, one that is not easy to spot, is the claim that because the amygdala is the fear center, activity there indicates that the defendant was experiencing high levels of fear*” (66). Authors who express this kind of criticisms assume that neuroscientists who promote the application of neuroscience methods in the forensic context, believe that neuro-scientific methods, and in particular neuroimaging, can be used in isolation (i.e., independently from the presence of any clinical symptoms) to claim insanity. In other words, the worry is that neuroscientists intend to replace the clinical assessment of insanity with neuroscience and neuroimaging methods. This criticism is based on a misinterpretation of the neuroscience position, a logical bias called “strawman fallacy.” Indeed, it provides a distorted version of the neuroscientist opinion, as neuroscientists are not claiming that brain imaging (or any other laboratory test) alone is sufficient nor that it should replace clinical interviews; rather, neuroscientists sustain that criteria for responsibility are and should remain based on clinical factors (22) and that brain scan exams may provide an objective support to the clinical diagnosis (15). Of note, a brain abnormality *per se* is not necessarily sufficient for determining insanity (15). For instance, the Italian serial killer Gianfranco Stevanin, despite a macroscopic traumatic lesion in the frontal lobe, was considered criminally liable because his ability to do otherwise was spared (15). Claiming that applying neuroimaging to insanity evaluation lacks of incremental validity above and beyond extant information (18, 21, 67) is simply untrue, as neurobiological evidence does contribute to solve diagnostic disputes and to obtain a more reliable diagnostic profile (68). As recently demonstrated, neuroimaging has indeed the potential to delineate also distinct and highly reproducible neuroanatomical subtypes within the same pathology (69). Thus, the incremental value of neuroscience lays in its usefulness to reduce the degree of intrinsic variability in insanity assessments (70). Of course, the relevance of neuro-scientific results in evaluating insanity may vary from case to case depending on individual features (71). Finally, any determinism should be strictly avoided (15).

### Neuroscientists Want to Infer Insanity From Brain Structure/Activation

“*A structural MRI reveals a brain defect in the frontal lobe, which is then used to justify the assertion that because of the defect, the person has impaired impulse control or impaired rationality*” (66). Similarly, “*Neuroimaging experts often posit direct connections between brain data and criminal actions, arguing that such connections form the foundation for an insanity defense*” (21). The above statements are two representative examples from recent literature of a major misconception of the utilization of neuro-scientific methods. As a matter of fact, neuroscientists are aware that brain imaging data cannot prove any direct causal connection between brain structure/function and criminal behavior. Indeed, we recently remarked that “*anatomo-clinical correlations can be assessed between brain region and cognitive functions but cannot be assessed between a brain region and criminal behavior, as there is no specific brain region involved in complex behaviors such as criminal violence*” (15). In other words, the anatomo-functional correlation should be assessed between neuroimaging data and neuropsychological test results for cognitive or behavioral functions that are relevant to comprehend the criminal act, such as impulse (dis)-control or (deficits in) moral reasoning. Furthermore, the causal link between behavioral alterations and the committed crime is another fundamental step in the evaluation of insanity (36). Thus, this criticism is affected by the “strawman” cognitive fallacy and violates each one of the four rules stated in recent *peer-reviewed* indications regarding the utilization of neuroimaging in court (15).

### Neuroscientific Techniques Do Not Comply With the Daubert Criteria

“*Many scientists have debated the relevance and admissibility of neuroimaging data to the insanity defense*” (21). In our view, this kind of criticism is too general and vague. For instance, it is not clear if it refers to structural or functional neuroimaging data. In addition, there are techniques that clearly comply with the Daubert criteria: one example is the MRI structural technique called Voxel Based Morphometry (VBM), which is used to analyze the structure of the brain in order to detect subtle brain changes that are not visible at gross inspection (72–74). These techniques, whose error rate is known both at the level of group and single individual analyses (75–77) and are fully accepted by the scientific community, unfortunately are sharply criticized when applied to the forensic field. Importantly, these criticisms are not directed to the techniques *per se*, but to the ways these techniques are believed to be utilized and their results to be interpreted. For instance, critical authors believe that VBM would be applied to find the neural basis of insanity and that any detected abnormality would be used to sustain insanity, regardless of the presence of clinical symptoms. On the contrary, neuroscientists clearly stated that VBM can only detect subtle brain abnormalities and that every inferences made on insanity is the result of a deductive reasoning based on stringent anatomo-clinical correlations (between the symptoms presented by the defendant and the anatomical localization of the abnormal brain region) and on the convergence of evidence, that of course are independent steps from VBM itself (15).

### The Study of the Brain Is Useless for Insanity Purposes as the Brain Is Dynamic in Nature

“*There is the problem of time: because people do not walk around wearing scanners, neuroimaging evidence presents information regarding brain structure or function after the fact*” (66). The above statement synthetizes the concern that brain imaging examinations are performed sometimes months, if not years, after the criminal facts have occurred. Although in most cases insanity assessments do take place a long time after the criminal acts, there are several aspects that need to be considered. In the first place, the time lag between the facts and the examination of the defendant applies to any component of the insanity assessment, including the psychiatric examination. As a matter of fact, psychiatric symptoms may rapidly change over time, both as a spontaneous evolution of the disorder and/or because of the treatments that are administered to the subject (78–80). Thus, establishing months later the “state of mind” of an individual at the time of the facts is typically an exercise based on assumptions and the evaluation of indirect pieces of evidence. Second, while it is undeniable that the brain is plastic, clinically relevant brain structural changes are obtained with exercise or rehabilitation, aging, drug or alcohol abuse or pathological processes (81–84). It is highly unlikely, if not impossible, that an individual's brain may undergo clinically significant modifications over a few months just because of the elapsed time, in the absence of any intervening brain accident or insult (e.g., infarct, head trauma, tumor). To clarify this issue with an example: let us assume that a MRI scan exam, for instance, shows a significantly smaller gray matter volume in the orbito-frontal regions, known to play a crucial role in impulse and behavioral control, in an individual who committed an impetus crime as compared to gender- and age-matched controls. That difference is real and it is irrelevant that the MRI scan had been performed a year after the facts, unless, of course, the subject in the meantime had suffered an accident that could have resulted in a loss of volume (e.g., severe head trauma, vascular events, resection of a tumor). Obviously, such a major event would be documented, especially given that the defendant would have been in custody since the very first days after the facts. An identical reasoning applies to functional brain data. Though certainly more susceptible to variability over time than structural data, measures of brain functional activation in response to specific tasks remain relatively stable within the same individual over time, unless there have been other interfering factors, such as assumption of psychotropic medications or illicit drugs, or pathologic events as mentioned above (85, 86). Thus, a brain anatomical alteration or a dysfunctional response in regions or circuits that modulate behavioral responses or cognitive functions relevant for insanity assessments, may be a valid element even if collected at distance of time after the facts. However, as stressful life events may affect brain morphology (87, 88), prolonged incarcerations theoretically could be associated with brain structural and functional changes. To what extent such changes may affect cortical regions that are crucial for self-determination and are therefore relevant in insanity assessments remains to be established.

On the other hand, the elapsed time may play a different role in the case that the brain lesion present at the time of the criminal act had been removed or repaired when the individual undergoes the insanity assessment. For instance, in a published case of acquired pedophilia, pedophilia emerged as a symptom of a *clivus* chordoma, a tumor of the notochord (89). While the defense consultants had the opportunity to examine the defendant prior to the surgical removal of the tumor, the *Periti* did not. Thus, they could not appreciate the variety of symptoms and signs that the consultants had detected at the time of the first examination. Eventually, the *Periti* concluded that the defendant was not insane at the time of the crime [for a discussion about the insanity assessment of this case, see also (16)].

### The Study of the Brain Is Problematic in Insanity Evaluation as Brain Images Should Be Interpreted

“*A functional or anatomical anomaly is interpreted as being a handicap only insofar as the behavior it may produce is considered such*” (18). We do agree that while results from neuro-scientific techniques (neuroimaging in particular) may inform on the biological basis for insanity, they cannot be themselves considered a proof of disability. This is necessary to avoid determinism. Neuro-scientific results should be interpreted taking into consideration the behavioral constellation of symptoms manifested by the defendant, with the advantage that neuro-scientific results cannot be malingered, unlike behaviors. For instance, a lesion in the frontal lobe can be the cause of a dis-inhibited and depraved sexual behavior conferring the base for insanity [see case 1 described in (15)], while a very similar lesion in the frontal lobe can be present in a defendant charged for murder during extreme sexual acts and feigning frontal-lobe induced dis-inhibition, thus leading to the decision of absence of insanity [see case 2 described in (15)]. Neuro-scientific results, including imaging data, may provide an objective contribution to solve controversies on insanity among experts, as described in a recent published paper (14). Unfortunately, clinical signs and symptoms may be interpreted in different ways [for instance, feigned or not feigned, hallucinations can be considered bizarre or not bizarre, a defendant who believes to have the right to decide who should die or live can be considered narcissistic or psychotic (52), etc.] leading to cognitive biases at the level of the conclusion of the Human Expert Performance model (45, 46) described in the first section of this paper. Neuroscientists who support the usefulness of neuroimaging are well-aware of its drawbacks and limitations: for this reason, we recently proposed objective indications on how to interpret neuroimaging results (15).

### The Inference Group to Individual Cannot Be Done

“*Science generalized, population level knowledge of a phenomenon does not necessarily provide an appropriate empirical foundation for making inferences about the instantiation of that phenomenon in any given individual*” (67); “*Virtually all brain scans rely on group data to determine the presence or absence of irregularities. The law, in contrast, typically relies on an idiographic approach, whereby each case is decided on its unique merits and facts*” (21). The first weakness of this criticism is that it does not distinguish between macroscopic and subtle brain alterations. Indeed, when gross brain alterations, such as brain tumors, traumatic injuries, advanced dementias, are considered, this statement is completely unfounded. In all these cases, which are the great majority, clinicians routinely make inferences at the level of the single individual in everyday clinical practice (i.e., when deciding whether to surgically remove the tumor, whether or not include a patient in a clinical trial, etc). Nevertheless, the use of neuroimaging has been criticized also in these cases (90). A distinct consideration is required in the case of subtle brain alterations, such as regional differences in neuronal density or fiber tracts, that can be detected only by performing a statistical analysis between group of patients and group of controls (91–94). Unlike neurological disorders, which usually are accompanied by brain alterations that can be easily revealed by routine laboratory exams, including tumors, strokes, atrophy or inflammatory processes, psychiatric disorders typically do not show gross morphological abnormalities. However, specific psychopathological features may indeed be associated with more subtle alterations in selected cortical or subcortical structures, as well as in neural circuits, that are devoted to emotional processing, modulation of aggressive behavior or moral judgment (11, 95–99). As they regard psycho-(patho)logical features, these abnormalities may very well be trans-diagnostic (100–102) and consistent across different ethnic groups (103). Though most of the above observations have been provided by group comparisons, in recent years researchers have aimed at enabling inferences to be made at the level of the individual subject (104–108). Further, the error rate of these single case analyses also were calculated in order to fulfill the Daubert criteria (75, 76). In addition, guidelines on how to correctly interpret these data were developed to prevent erroneous conclusions (15). Noteworthy, the scientific community is working to improve translation of neuroimaging findings to the clinical settings through the implementation of algorithms able to identify brain abnormalities in individual patients. Indeed, eight tools already have been released freely online to support clinical diagnoses [for a review see (53, 109)]: clinicians can upload the MRI scans of an individual patient; an automated and validated algorithm will analyze the brain to detect potential structural alterations and a detailed report will be generated and sent to the clinician. Finally, recent advances have proposed a normative modeling in neuroimaging analyses of psychiatric disorders (110, 111). According to this approach, the degree to which each individual brain deviates from the normative pattern is calculated so that these deviations can be mapped in each individual and psychopathology can be conceptualized as deviation from normative patterns (110–113). In other words, the normative modeling provides a way to quantify and characterize the manner in which the brain of different individuals deviates from the expected normal brain (110, 113). Authors clearly declared that this approach cannot indicate directly whether the obtained deviations are clinically relevant (110), and for this reason neuroimaging results should assume clinical significance only when associated with clinical symptoms. In summary, group to individual inferences are possible even when structural abnormalities are not grossly evident and are revealed by rigorous and sophisticated statistical analyses.

### Neuroimaging Are Useless as There Is no Known Neural Basis for Insanity

“*Large-scale normative imaging data on individuals found insane have yet to be collected (…) this means that data are not sufficiently advanced to bear direct relevance to insanity determination*” (21). Insanity is not a specific syndrome or disease that can be investigated as a unified entity, but rather may derive from a variety of diseases with a different etiology. For instance, insanity may be due to dementias, brain tumors, psychoses, traumatic brain injuries. Of course, the brain regions involved in these pathologies may widely differ, thus it is highly unlikely to find a common neural substrate invariably shared by individuals found to be insane. A further complication is that insanity is a condition defined by the law, not a medical diagnosis. Specifically, it is up to the judge to ultimately decide whether or not the defendant is insane: such a decision not necessarily reflects the expert conclusions nor, as discussed above, the experts from the different sides may necessarily agree on the presence of insanity in the first place. Thus, a search for the neural basis of insanity as such would be affected by major conceptual caveats, as the very same individual could be considered completely insane by an expert and fully capable by another one. Rather, a promising strategy in this direction is represented by the search for the neural basis that may underlie abnormal/antisocial behaviors in specific categories of offenders including rapists (97), batterers (96, 114), or murderers (11, 98). Unfortunately, biased by the logical fallacy called circular reasoning, the above studies are criticized as well, as they are believed to look for a neural basis of insanity (21).

### Neuroimaging Data in the Absence of Clinical Symptoms Have Been Used to Sustain Forensic Conclusions

Criminal cases in which brain imaging was misused, such as the notorious case of Weinstein, in which the defense attorneys sustained a decreased culpability because of the presence of a congenital arachnoid cyst, disregarding the essentially complete lack of any psychiatric symptoms or behavioral abnormalities in the defendant, are brought up by critical authors to oppose the introduction of neuroimaging in the forensic context (66, 115). To claim that neuroimaging as a whole is useless or dangerous, by citing selected sporadic cases of misuse of neuroimaging findings, is a logical bias. Indeed, neuroscientists agree that in the Weinstein case brain imaging results were improperly interpreted, as there were neither behavioral symptoms nor anatomo-clinical correlations nor any causal link. In other words, in the Weinstein case all the rules for the correct use of brain imaging in court proposed by recent guidelines (15) were violated.

In many cases, however, brain scans have provided an instrumental contribution to support insanity (89) and to disentangle problematic clinical diagnoses (14). For instance, in the famous case of Hinckley, who shot President Reagan (United States v. Hinckley, 525 F. Supp. 1324, 1982), brain computerized tomography scans revealed enlarged ventricles, thus supporting the diagnosis of schizophrenia rather than personality disorder, which were the two diagnoses sustained by the experts working for the two adversarial sides, respectively.

To conclude, overall it appears that the criticisms raised against the adoption of neuro-scientific methods, in particular neuroimaging, in the forensic process are based on erroneous assumptions and mis-representations of the role of neuroscience methods in court. In line with previously published analyses (15), here we explained why these criticisms are scientifically ungrounded and are affected by logical/cognitive fallacies.

## How Neuroscience Can Improve Insanity Evaluation

In criminal trials, the judge and juror decisions should be made in accordance with the “*beyond any reasonable doubt*” principle. In the first section (“The intrinsic unreliability of clinical assessment of mental disorders and insanity”) we have discussed the reasons why a psychiatric diagnosis based merely on the clinical observation is intrinsically prone to errors. Some criminal field experts propose to couple unstructured clinical interviews with results from neuro-scientific evaluations as a strategy to overcome this critical aspect, improving reliability in insanity evaluations and reducing controversies. In the second section (“Criticisms to the neuro-scientific approach”), we presented the major criticisms to the introduction of neuroscience in court and we explained why they do not provide any scientifically valid basis to reject the utilization of the neuro-scientific approach as a whole in the field of forensic psychiatry. In this last section, we will discuss how the neuro-scientific approach can be usefully implemented to improve insanity evaluations. It is, however, of the utmost importance to specify in advance what neuroscientists do not claim, in order to avoid any risk for further misinterpretation of these opinions.

### What Neuroscientists Do Not Claim

#### Neuro-Scientific Methods Aim to Change the Rationale Underlying the Determination of Criminal Liability

Neuroscientists do not claim that the institution of criminal responsibility should be revolutionized. The determination of responsibility and insanity is and shall remain based on the assessment of behavioral symptoms in the particular legal context the defendant is evaluated (15, 22).

#### Science Is Devoid of Errors

Neuroscientists are aware that the scientific reasoning is falsificationist; thus, their *modus operandi* is to try to falsify their own hypotheses. Furthermore, neuroscientists are aware that all human beings are prone to both errors and cognitive biases and therefore they study how to recognize and minimize these issues (40, 46, 47).

#### Neuroscience Alone Is Enough

Neuroscientists are not claiming that neuro-scientific methods (including in particular neuroimaging) should be used in isolation, independently from clinical evaluations. On the contrary, recent guidelines emphasize that neuroimaging findings should always be coupled with clinical findings (15). Indeed, when neuroimaging is used in the absence of clinical symptoms, cognitive fallacies emerge in the interpretation of the results.

### What Is the Usefulness of the Introduction of Neuro-Scientific Methods in Criminal Trials?

As already discussed above, neuroscience does not intend to introduce a “brain-based” concept of insanity. Rather, neuroscientists wish to contribute to develop a scientific way to evaluate insanity with the ultimate aim to reduce controversies and errors in criminal trials, due to both the intrinsic unreliability of insanity assessments and the unconscious proneness to logical bias common to all human beings. How does neuroscience pursue this aim?

#### Implementing a Rigorous Scientific Logic in Insanity Assessment Procedures

According to the scientific logic, which is adopted by both medicine and law, the data used to create a hypothesis cannot be used also to also confirm or disprove the same hypothesis. By strictly adopting scientific logic within the insanity assessment procedure, expert consultants should first formulate their diagnostic hypothesis by using unstructured interviews and then they should validate their hypothesis by using complementary methods: analysis of clinical history, neuropsychological tests, psycho-pathological tests, laboratory examinations. This is exactly what physicians do when they examine a patient: search for independent and objective data to confirm (or disprove) their diagnostic suspect.

#### Looking for Converging Pieces of Evidence

As clinical pieces of evidence, based on patients self-report only, suffer from low inter-rater reliability and are therefore questionable (31–33), a diagnostic hypothesis should be corroborated by findings coming from independent, possibly trans-disciplinary, sources (16). For instance, a clinical diagnosis can be supported by psychopathological data coming from self-reported questionnaire that are less prone to malingering, including the Minnesota Multiphasic Personality Inventory-2 (MMPI-2) (116).

#### Decrease the Potential Impact of Cognitive Biases on Decisions

The presence of independent but converging pieces of evidence, obtained using a trans-disciplinary approach, will help to mitigate the influence of cognitive biases (14, 42, 44–46, 50), as this approach would make it much more difficult to adopt logical fallacies to rule out multiple and consistent pieces of evidence (16, 17, 117).

Neuroscientists are aware that science is not free from limitations and errors; indeed, progress stems from this fundamental awareness. Results from psychopathological, neuropsychological and neuroimaging exams may turn out to be fallacious as much as psychiatric interviews. However, in a context where decisions should be reached “*beyond any reasonable doubt*,” the 53% the error rate of unstructured psychiatric interview (31, 33) does not appear to be acceptable. Then, it is certainly preferable to base any insanity decision on converging pieces of evidence, each of them obtained by methodologies that, though potentially prone to errors themselves, all provide coherent results leading to the same conclusion. In sum, we suggest that unstructured interviews should be considered the necessary but not sufficient starting step for the insanity assessment: it is pivotal to formulate a diagnostic hypothesis that should then be corroborated by a multidisciplinary assessment.

Thus, the neuro-scientific multimodal and trans-disciplinary approach aims to minimize the risk of cognitive biases and to decrease controversies by using a scientific falsificationist logic and by following the convergence of evidence principle, in order to improve the overall reliability of the insanity evaluation process. The final goal is to make the decision on insanity less questionable, to reduce the duration of criminal trials and to increase the likelihood for every defendant to receive a fair punishment. In many instances, individuals may benefit more from a medical treatment measure rather than from an exclusively retributive punishment involving incarceration without treatment. Thus, ensuring a fair and unbiased insanity evaluation is not only a juridical need, but also a medical and ethical requirement.

## Discussion

The necessity to improve the inter-rater reliability in the procedures adopted to answer juridical questions is a timely topic. The neuro-scientific approach, having a multimodal and trans-disciplinary nature, can help to find a fair solution to cases which may be particularly prone to judicial error, as it occurs with insanity evaluations. Criminal systems, jurors and judges should not neglect the relevant drawbacks of the classical approach to insanity assessment based merely on unstructured interviews. This insanity assessment procedure is intrinsically biased by the low inter-rater reliability of psychiatric diagnoses (32) and by the presence of cognitive biases in psychiatric consultants (40, 47). While certain ethical aspects also are involved (118), the neuro-scientific approach offers a strategy to mitigate these problems, as it adopts methods with significantly lower error rates than the classical assessment. Thus results from unstructured clinical interviews should be integrated with data from standardized and objective neuropsychological and psychopathological tests (31, 33) and from laboratory exams (9, 22, 119) and interpreted in respect of the convergence of evidence principle (14, 120).

Strategies to improve the reliability of insanity evaluations should be encouraged within the forensic and legal systems. Perital questions to the consultants should be formulated in ways that are less prone to subjective interpretations. For instance, consultants could be asked to use methods that are characterized by a lower risk of bias. This may be a critical step to enhance diagnostic accuracy, which is a fundamental component in insanity assessments. Consultants also may be required to provide the psychometric indices (i.e., validity and inter-rater reliability) of the methods and instruments they intend to use for the insanity evaluation, that is, unstructured interviews, structured interviews, questionnaires, psychopathological and neuropsychological tests. This would help judges and jurors to evaluate the relative contribution of each technique/test to the insanity judgment. Finally, consultants should be asked to provide converging evidence in support of their conclusions from all the methodologies they used. This would greatly contribute to decrease the diagnostic uncertainty and the probability to commit errors.

Psychiatric consultants are advocated to pay attention to the methodologies they rely upon. They should be aware of the strengths and limitations of each method they adopt. This awareness should be considered a strength, as this will allow the identification of new strategies to solve the problem, as for instance by adopting the convergence of evidence approach. A policy of problem denial inhibits the improvement of evaluation processing and discourages the critical evaluation of actions. Consequently, a setting where problems are regarded as an incentive for improvement is required (40). Second, the reasons why a certain psychiatric diagnosis is formulated should be clearly stated. In other words, as the DSM-5 provides specific diagnostic criteria for each diagnosis, the criteria that are present or absent in the defendant should be clearly stated. This would favor the scientific debate in court and would prevent discussions about ungrounded diagnoses. Third, consultants should never neglect the functional analysis of the crime; that is, they should explain how a given psychiatric diagnosis is causally linked to the criminal behavior. Fourth, the consultant conclusions on insanity should be logically sustained to enhance transparency.

Attorneys and lawyers can promote the scientific basis of criminal trials as well. First, they can ask their consultants to identify the potential presence of logical fallacies in the thesis of the opposite consultants. This will enable them to inform the judges on the presence of cognitive biases and, eventually, will open the door for appeal. Second, attorneys should prepare the opponent's cross-examination focusing also on the methodological aspects: validity, reliability, accuracy of the methods they have applied. Too general answers as “*everyone uses this method*” or “*I have done in this way for decades*” should not be accepted, as they are apodictic and unscientific. This would help to improve the scientific nature of insanity evaluations and to reduce their still high error rate.

In conclusion, neuroscience does not intend to replace the classical approach to the insanity assessment procedure, but rather aims to make it more reliable, less prone to cognitive biases so to reduce uncertainty and controversies. What is not clear is why this process, which has been welcomed by all the other medical braches (e.g., neurology welcomed the advent of MRI to support the diagnosis of Alzheimer's and multiple sclerosis), is still considered worrisome in forensic psychiatry. Indeed, the major limitation to the introduction of a neuro-scientific approach into the legal settings does not come from the judges and jurors, but from scientists themselves who, in accordance with the denial cognitive bias (40, 48), prefer to maintain a disputable methodology rather than to improve the overall procedure by adopting a multidisciplinary approach based on the convergence of evidence principle. This problem appears to be specific for forensic psychiatry, while it is not present in clinical psychiatry, as demonstrated by the constant growing of studies that search for potential ways to enhance translational application of neuro-scientific findings into clinical psychiatric settings (53, 110, 119, 121–124).

## Data Availability Statement

The original contributions presented in the study are included in the article/supplementary material, further inquiries can be directed to the corresponding author/s.

## Author Contributions

All authors listed have made a substantial, direct and intellectual contribution to the work, and approved it for publication.

## Conflict of Interest

The authors declare that the research was conducted in the absence of any commercial or financial relationships that could be construed as a potential conflict of interest.
